# Suicidal ideation among Lebanese adolescents: scale validation, prevalence and correlates

**DOI:** 10.1186/s12888-020-02726-6

**Published:** 2020-06-15

**Authors:** Melissa Chahine, Pascale Salameh, Chadia Haddad, Hala Sacre, Michel Soufia, Marwan Akel, Sahar Obeid, Rabih Hallit, Souheil Hallit

**Affiliations:** 1 Faculty of Medicine and Medical Sciences, Holy Spirit University of Kaslik (USEK), Jounieh, Lebanon; 2 INSPECT-LB: Institut National de Santé Publique, Épidémiologie Clinique et Toxicologie, Beirut, Lebanon; 3 Faculty of Pharmacy, Lebanese University, Hadat, Lebanon; 4 Faculty of Medicine, Lebanese University, Hadat, Lebanon; 5 Research Department, Psychiatric Hospital of the Cross, Jal Eddib, Lebanon; 6 Université de Limoges, UMR 1094, Neuroépidémiologie Tropicale, Institut d’Epidémiologie et de Neurologie Tropicale, GEIST, 87000 Limoges, France; 7 Drug Information Center, Order of Pharmacists of Lebanon, Beirut, Lebanon; 8 School of Pharmacy, Lebanese International University, Beirut, Lebanon; 9 Faculty of Arts and Sciences, Holy Spirit University of Kaslik (USEK), Jounieh, Lebanon

**Keywords:** Suicidal ideation, Adolescents, Child abuse, Alcohol use disorder, Bullying, Internet addiction, Impulsivity, Social fear

## Abstract

**Background:**

In addition to the unstable political situation, Lebanon had experienced a cycle of wars, local armed conflicts, terrorist attacks, and crises (lack of clean water, recurrent power failure, and waste mismanagement, in addition to the growing number of unemployed people, as the number of Syrian refugees has dramatically increased, and led to competition for jobs with locals. All these factors make the Lebanese population prone to mental disorders, particularly suicide, without clear management policies. This study aims to validate the Columbia-Suicide Severity Rating Scale (CSSRS), and determine the prevalence of suicidal ideation and associated factors among a Lebanese nationally representative sample of adolescents from 9th to 12th grades.

**Methods:**

Participants were 1810 adolescents who enrolled in this cross-sectional study (January–May 2019), using a proportionate random sample of schools from all Lebanese Mohafazat. The Columbia-Suicide Severity Rating Scale was used to screen for suicidal ideation.

**Results:**

The results showed that 28.9% had some type of suicidal ideation [95% CI 26.7–31.1%]. The CSSRS items converged on a one-factor solution, accounting for a total of 85.40% of the variance (α_Cronbach_ = 0.966). Higher psychological abuse (Beta = 0.041), child physical abuse (Beta = 0.030), alcohol dependence (Beta = 0.062), social fear (Beta = 0.028), victimization/bullying score (Beta = 0.028), impulsivity (Beta = 0.028) and internet addiction (Beta = 0.010) scores were significantly associated with higher suicidal ideation. Same applies to adolescents whose parents are separated compared to living together (Beta = 0.992) and in females compared to males (0.311). On another hand, a higher age (Beta = − 0.182) was significantly associated with lower suicidal ideation.

**Conclusion:**

This study provides insights about suicidal ideation among Lebanese adolescents and related risk factors, such as child psychological and physical abuse, alcohol use disorders, social fear, bullying and victimization, impulsivity, and internet addiction.

## Background

### Suicidal ideation prevalence and risk factors

Adolescence is a period of transition in the life of a human during which people acquire physiological and psychosocial maturity. The changes in this phase make the adolescents vulnerable, as they become more autonomous in their choices and seek to be independent from their families. During this phase, adolescents develop knowledge and skills, learn how to cope with their feelings and relations, acquire the ability to interpret situations, and become able to confront experiences and face different problems in their lives [1]. Adolescence is marked by a significant prevalence of psychiatric disorders and risk of suicide. According to the WHO (World Health Organization), 16 people per 100,000 die from suicide each year (or one death every 40 s), and in 2020, this mortality rate is expected to increase to one every 20 s [2]. In 2016, suicide was the 18th cause of death worldwide and the second principal cause of death among those between 15 and 29 years old [2].

Worldwide, risk factors of suicide include victimization (bullying, sexual harassment), poor mental health related to depression, social phobia, anxiety, impulsivity, alcohol use disorder, child abuse, lack of parental understanding [3], and internet addiction [4].

Bullying, defined as a repeated aggressive behavior intending to harm a victim, can be verbal, physical, relational, or emotional [5]. Many mental health consequences can result from peer victimization, such as anger, sadness, anxiety, depression, self-harm, suicidal ideations and attempts [6]; also, suicidal ideation and peer victimization present a dose-response relationship: the risk for suicide increases with bullying [7].

Independent from bullying, depression is common during adolescence and increases post-puberty, with a 12-month median prevalence of 4–5% worldwide [8]; it can be due to genetic factors [9], physiological changes [10], and environmental and psychosocial factors, such as unpleasant events, and chronic adversity [11]. Depression has been strongly associated with suicide among adolescents [12]. Indeed, more than half of those who attempted suicide reported being depressed at the time of the attempt [13]. Nevertheless, it is believed that depression alone is not sufficient to trigger suicide among those with suicidal ideation [14].

As for phobias and anxiety disorders, they are common in adolescents and associated with many comorbidities such as depression, alcohol use, and behavioral disorders [15]: it has been demonstrated that adolescents with anxiety disorders and major depressive disorder are at increased risk of suicide.

Impulsivity, defined as the inability to think and measure the consequences before acting [16], is the immediate stage before suicide attempts when it is at increased levels [17]. Moreover, some studies have shown that impulsivity was positively related to suicide in young adults, with an even stronger association in the younger population [18–20], contrary to other research that could not find a significant correlation between the two [21, 22].

Furthermore, alcohol consumption in adolescents, a public health concern in the US [23], has been positively associated with higher levels of suicidal ideations [24, 25]. Some studies have established a link between age at first alcohol use and some health risk behaviors [26, 27], including higher suicidal ideation among adolescents [28].

Also, child abuse, whether psychological, emotional, physical, or sexual, is associated with suicide [29]. Indeed, child physical abuse showed to induce a higher risk of suicidal ideation in both adolescence and adulthood [30].

Finally, with advances in technology and communication, the pathological use of the internet has emerged as a new factor associated with suicide [4], becoming a public health issue nowadays, with its prevalence increasing from 0.8 to 26.7% worldwide [31]. Adolescents with internet addiction are at increased risk for self-injuries, and suicidal ideations and attempts [32–35], due to higher feelings of loneliness in this population [36].

### Suicidal ideation in the Arab world

Data on suicidality are limited in the Arab World, but a study from the Gulf countries on adolescents between 10 and 19, showed that suicide was among the top 5 causes of death in this age group [37]. Although spirituality, religiosity, and religion strongly permeate the Arab world, they might not protect against suicidal ideation; they can only decrease suicidal attempts and behaviors since both Islam and Christianity prohibit suicide and consider it as a sin [38]. Furthermore, the topic may be considered taboo in the region [3], and some may believe in the hypothesis that asking about suicidal ideations might generate them [39].

### Suicidal ideation in Lebanon

Mental health problems in the general population in Lebanon are as high as in Western European countries where mental health issues are frequent, like Belgium, France, Germany, Italy, the Netherlands, and Spain [40], but the number of affected individuals left untreated remains high [40]. In 2003, a study revealed that approximately 50% of the Lebanese population was threatened by traumatic events due to conflicts [41]. In addition to the unstable political situation, Lebanon had experienced a cycle of wars, local armed conflicts, and terrorist attacks [42]. Many crises, such as the lack of clean water, recurrent power failure, and waste mismanagement, overwhelmed the country as well [28, 42]. All these problems are coupled with the growing number of unemployed people, as the number of Syrian refugees has dramatically increased, and led to competition for jobs with locals [43], and lack of public awareness concerning mental disorders, as well as cultural sensitivity to the subject that remains taboo, especially when treatment is needed. All these factors make the Lebanese population prone to mental disorders, particularly suicide, without clear management policies [44]. A study conducted in 2005 on a sample of 5038 Lebanese adolescents, showed that around 16% of Lebanese adolescents thought of suicide, and addressed risk factors for suicidal ideations [3], i.e., poor mental health, alcohol use and drug abuse, victimization, and lack of parental understanding [3], but did not include significant factors, such as tobacco use (cigarette/waterpipe smoking), internet addiction, impulsivity, and child abuse.

### Screening of suicidal ideation

The screening for suicidal ideation and behavior is performed using the Columbia-Suicide Severity Rating Scale (C-SSRS) [45], an internationally approved scale for the assessment of suicidality and one of the most used in clinical trials, part of the Treatment of Adolescent Suicide Attempters (TASA) study that assesses suicidal risk [46]. This scale is also used in different settings to distinguish suicidal from non-suicidal self-injurious behavior, provide definitions of these two entities, and measure the full range of suicidal behavior and suicidal ideation [46–49]. It also estimates their severity and intensity over indicated periods and defines the type and lethality of suicidal behavior [46, 50], and selected items predict the risk of suicide, such as preparatory activity [50]. It has been translated into 125 specific country languages with 75 countries translations include the Arabic language for Egypt, Jordan, Morocco, Saudi Arabia, Tunisia, and the Lebanese Arabic language [51]. However, no psychometric validation of the scale was performed in Lebanon.

### Objective

Based on the abovementioned facts, this study aims to validate the Columbia-Suicide Severity Rating Scale in Lebanon and determine the prevalence of suicidal ideation and potential risk factors among a Lebanese nationally representative sample of adolescents from 9th to 12th grades. Factors to be assessed include poor mental health (loneliness, worry, sadness, or hopelessness), alcohol use disorder, victimization (bullying, sexual harassment), and lack of parental understanding, social phobia, depression, impulsivity, child abuse, and internet addiction. The results of our study could be a gateway to new studies to assess other factors that may be associated with suicidal thoughts, such as genetic factors. They will also help issue new recommendations on how teens should deal with daily life stress and how parents should provide emotional support to their children.

## Methods

### Participants

This cross-sectional study was conducted between January and May 2019, using a proportionate random sample of schools from all Lebanese Mohafazat (Beirut, Mount Lebanon, North, South, and Bekaa). A total of 18 private schools was contacted; two refused to participate. Those who accepted were located as follows: 4 in Beirut, 2 in South Lebanon, 6 in Mount Lebanon, 2 in North Lebanon, and 2 in the Bekaa. Of 2000 questionnaires distributed, 1810 (81.0%) were filled and collected back. Participants were randomly selected from each school and were between 14 to 17 years old. Students were free to participate in the study or not and received no financial compensation for their participation. This study excluded illiterate adolescents between age 14 and 17 years old, those with a mental disability or cognitive impairment, those who have a chronic psychiatric disease, or any self-reported suicidal attempt, and adolescents who refused to participate in the study. The same methodology is described in other papers [52, 53].

### Minimal sample size calculation

Ten observations for each scale item were found necessary according to Comrey and Lee in order to properly validate a scale [54]. The C-SSRS scale is comprises six questions, therefore, 60 patients were needed, at least, for the conduction of the exploratory factor analysis.

### Questionnaire

The questionnaire was in Arabic, the native language of Lebanon, and required approximately 60 min to complete. Participants filled out the questionnaire in the classrooms to avoid parental influence while answering the questions. Completed questionnaires were handed back to the team and sent for data entry.

The first part of the questionnaire consisted of the sociodemographic questions (i.e. age, gender, smoking status, parents’ status), in addition to other characteristics, including the Body Mass Index (BMI), the household crowding index, and the Total Physical Activity Index. The BMI (kg/m^2^) was calculated based on self-reported height and weight of the participants. The household crowding index was calculated by dividing the number of persons living in the house and the number of rooms in the house, excluding the bathroom and the kitchen [55], and the Total Physical Activity Index was calculated by multiplying the intensity, duration and frequency of daily activity [56].

The second part of the questionnaire included the following scales:

#### Columbia-suicide severity rating scale (C-SSRS)

It is a six-item tool used to assess suicidal ideation and behavior. Questions 1 to 5 evaluate suicidal behavior over the past month, while question 6 evaluates it over the respondent’s lifetime and past 3 months. Answering “yes” to any of the 6 questions indicates the presence of suicidal ideation and the need for referral to a specialist [57] (in this study, α_Cronbach_ = 0.966, compared to 0.946 in previous studies [46, 58]). Dr. Kelly Posner gave us the permission to use the Arabic version of the scale.

#### The Illinois bully scale (IBS)

The Illinois Bully Scale is a 16-item research-validated tool used to measure bullying and victimization by directly surveying students. It is divided into two sections: a bullying scale that measures student involvement in bullying (questions 1–9) and a victimization scale that measures the extent to which students have been bullied (questions 10–16). Both measures are combined to yield a bullying and victimization score [59]. Greater scores indicate higher bullying and victimization (in this study, α_Cronbach_ = 0.975, compared to 0.87 in a previous study [60]).

#### Internet addiction test (IAT)

The valid Arabic version [61] comprising 20 items was used. It is scored on a 6-point Likert scale from 0 (does not apply/never) to 5 (always applies). Greater scores indicating higher internet addiction (in this study, α_Cronbach_ = 0.925, and ranged between 0.891 [62] and 0.914 in other studies [63, 64]).

#### The adolescent depression rating scale (ADRS)

This 10-item scale was developed to screen for depression among adolescents, with questions rated as yes/no. Higher scores indicate higher levels of depression [65]. The Cronbach’s alpha for this scale in this study was 0.940, compared to 0.78 in the original one [65].

#### The alcohol use disorders identification test (AUDIT)

The 10-item self-report version of the AUDIT and standard drinks measures was used [66]. This scale has been validated in a sample of Lebanese adolescents. A score of 8 or more indicated hazardous alcohol drinking (in this study, α_Cronbach_ = 0.960, compared to 0.93 in a previous paper [67]).

#### Liebowitz social anxiety scale (LSAS)

This self-report scale version was used to help identify social anxiety [68, 69]. It features 24 items divided into two subcategories (13 questions relate to performance anxiety and 11 to social situations), scored on a 4-point Likert scale from 0 to 3. The maximum score (144 points) reflects a very severe social phobia. In this study, the Cronbach’s alpha values were 0.969 for the total score, and 0.952 and 0.951 for the fear and avoidance subscales, respectively. In another study, it was 0.79 [70].

#### BARRAT impulsiveness scale (BIS-11)

This scale is used to assess the personality/behavioral characteristics of impulsiveness. It is a self-report 30-item tool divided into 3 major subcategories: a) motor impulsivity that assesses the tendency to act on the spur of the moment, b) non-planning impulsivity that measures careful thinking and planning for the future, and c) attentional impulsivity that assesses task-focus thoughts and cognitive complexity. Items are scored on a 4-point Likert scale from 1 (absent or rare) to 4 (present or extreme). A score of 72 and above indicates a high level of impulsiveness [71]. The Cronbach’s alpha for this scale in this study, was 0.921, compared to 0.77 in previous papers [72, 73].

#### Child abuse self-report scale (CASRS)

This 38-item validated tool is divided into 4 categories of child abuse and neglect: psychological (14 items), neglect (11 items), physical (8 items) and sexual abuse (5 items). It is scored on a 4-point Likert scale (0 = Never, 1 = Sometimes, 2 = Most often, 3 = Always) [74]. A score of 0 indicates no abuse or neglect, while a score of 3 reflects severe abuse or neglect, with high scores indicating more childhood abuse. The Cronbach’s alpha values for each subscale in this study were as follows: psychological (0.973), neglect (0.971), physical (0.966) and sexual (0.954). The reliability of the total scale in the Iranian study was 0.92 [74].

### Translation procedure

One health professional, knowledgeable in the scales’ terminology, whose mother tongue is Arabic and is fluent in English, translated the scales (except the C-SSRS, IBS, IAT and AUDIT scales) from English to Arabic. Another health professional, whose mother tongue is English and is fluent in Arabic, translated the Arabic version back to English. Both translators were aware of the study purpose. The two English versions were compared at the end of this procedure, by experts, in order to discriminate any variations. Translators were responsible for updating the translated versions once those variations were communicated to them. Discrepancies between the two English versions were resolved by consensus.

### Statistical analysis

Data analysis was performed using the SPSS software version 23. Since our sample included more than 100 participants, the data was considered normally distributed, whereby non-normal distributions have no significant consequences in the case of samples greater than 100 [75]. Missing data constituted < 10% of the total database and therefore was not replaced. Descriptive analyses were done using counts and percentages for categorical variables and mean and standard deviation for continuous measures. Reliability was checked using Cronbach’s alpha values for different factors and the total scale. The Student t-test was used to compare continuous variables between two groups, and Pearson correlation was used for linear correlation between continuous variables. For categorical variables, the chi-square and Fisher exact tests were used. The Student t-test was used to compare the means of 2 groups, and the ANOVA was used to compare three or more groups. A stepwise linear regression was performed taking the suicidal ideation score as the dependent variable. Variables that showed a *p* < 0.1 in the bivariate analysis were included in the model to eliminate potentially confounding factors as much as possible. Significance was set at *p* < 0.05. A factor analysis was initiated using the “principal component analysis” technique to confirm the legitimacy of the construct of the C-SSRS in our sample; no rotation was selected since the extracted factors were found to be meaningfully associated. The Kaiser-Meyer-Olkin (KMO) measurement of sampling adequacy and Bartlett’s sphericity test were appropriate. The factors retained corresponded to Eigenvalues greater than one.

## Results

The sociodemographic characteristics of the participants are summarized in Table 1. The mean age was 15.42 ± 1.14 years, with 53.3% females, 74.1% nonsmokers, and. The results also showed that 11.9% of the adolescents had separated/divorced parents, and 28.9% had some type of suicidal ideation [95% CI 26.7–31.1%].

**Table 1:** Sociodemographic characteristics of the sample population

	Frequency (%)
**Gender**	
Male	844 (46.7%)
Female	963 (53.3%)
**Parents status**	
Living together	1581(88.1%)
Separate	213 (11.9%)
**Smoking status**	
Yes	468 (25.9%)
No	1342 (74.1%)
	**Mean ± SD**
**Age (years)**	15.42 ± 1.14
**Body Mass Index (kg/m2)**	21.95 ± 4.21
**Household crowding index**	1.01 ± 0.64

### Factor analysis of the suicidal ideation score

The total sample (*n* = 1810) was used for the factor analysis; all items of the C-SSRS were extracted and yielded a one-factor solution with Eigenvalues > 1 (variance explained = 85.40%; KMO = 0.877; Bartlett’s sphericity test *p* < 0.001; α_Cronbach_ = 0.966). The components generated are summarized in Table 2.

**Table 2:** Principal component analysis results of the Columbia–Suicide Severity Rating Scale

Question	Item	Loading factor
Active Suicidal Ideation with Some Intent to Act, without Specific Plan	4	0.950
Non-Specific Active Suicidal Thoughts	2	0.936
Active Suicidal Ideation with Specific Plan and Intent	5	0.934
Active Suicidal Ideation with Any Methods (Not Plan) without Intent to Act	3	0.925
Suicidal behavior (actual attempt, interrupted attempt, aborted attempt)	6	0.919
Wish to be Dead	1	0.878

### Convergent validity

Higher suicidal ideation was significantly associated with higher depression (*r* = 0.144) and higher social fear (*r* = 0.454) and avoidance (*r* = 0.330).

### Bivariate analysis

The results of the bivariate analysis are summarized in Table 3. A higher mean of suicidal ideation score was significantly found in adolescents whose parents are separated compared to those whose parents live together (2.86 vs. 0.75) and in females compared to males (1.22 vs. 0.77). Moreover, a higher suicidal ideation score was significantly associated with higher scores of bullying victimization, cigarette dependence, waterpipe dependence, social fear and avoidance, internet and alcohol addiction, depression, psychological abuse, neglect, and physical and sexual child abuse. Higher age and physical activity were significantly associated with lower suicidal ideation.

**Table 3:** Bivariate analysis taking the Suicidal ideation score as the dependent variable

	Suicidal ideation score	***P*** -value
	Mean ± SD	
**Gender**		
Male	0.77 ± 1.61	**< 0.001**
Female	1.22 ± 1.99	
**Parents status**		
Living together	0.75 ± 1.57	**< 0.001**
Separate	2.86 ± 2.41	
	**Correlation coefficient**	***P*-value**
Age	−0.068	**0.006**
Victimization/Bullying score	0.289	**< 0.001**
Liebowitz – Fear	0.454	**< 0.001**
Liebowitz – avoidance	0.330	**< 0.001**
Internet addiction score	0.209	**< 0.001**
AUDIT score	0.481	**< 0.001**
House crowding index	−0.047	0.061
Physical activity score	−0.077	**0.003**
Depression total score	0.144	**< 0.001**
Psychological abuse scale	0.527	**< 0.001**
Child abuse neglect scale	0.132	**< 0.001**
Child abuse physical scale	0.473	**< 0.001**
Child abuse sexual scale	0.419	**< 0.001**
Impulsivity	0.185	**< 0.001**

*AUDIT* Alcohol Use Disorders Identification Test, *SD* Standard deviation; numbers in bold indicate significant *p*-values

### Multivariable analysis

A linear regression, taking the suicidal ideation score as the dependent variable, showed that a one-point increase in the psychological abuse, child physical abuse, alcohol dependence, social fear, victimization/bullying score, impulsivity, and internet addiction scores would significantly be associated with higher suicidal ideation score by 0.041, 0.03, 0.06, 0.028, 0.028, 0.028, and 0.01 points respectively. Having parents who are separated compared to living together would be associated with higher suicidal ideation scores by 0.992 points, while the female gender would be associated with higher suicidal ideation scores by 0.311 points. Moreover, each additional age increase by 1 year would significantly be associated with a lower suicidal ideation score by 0.182 points (Table 4).

**Table 4:** Multivariable analysis. Linear regression taking the Suicidal ideation score as the dependent variable

	Unstandardized Beta	Standardized Beta	*p*-value	Confidence Interval	
				Lower	Upper
Psychological abuse	0.041	0.238	< 0.001	0.031	0.051
Child abuse physical	0.030	0.100	< 0.001	0.013	0.047
AUDIT score	0.062	0.273	< 0.001	0.051	0.073
Liebowitz fear score	0.028	0.250	< 0.001	0.023	0.033
Parents status (separated vs living together^a^)	0.992	0.181	< 0.001	0.767	1.217
Age	−0.182	−0.112	< 0.001	−0.242	−0.121
BARRAT total score	0.028	0.126	< 0.001	0.018	0.038
Gender (female vs male^a^)	0.311	0.082	< 0.001	0.167	0.455
Victimization Bullying score	0.028	0.108	< 0.001	0.016	0.041
IAT score	0.010	0.096	< 0.001	0.005	0.014

Variables entered in the model: age, gender, parents status, BARRAT score, Liebowitz –fear score, Liebowitz – avoidance score, AUDIT score, LWDS-11 score, FTND, depression, Psychological abuse scale, Child abuse neglect scale, Child abuse physical scale, Child abuse sexual scale

^a^Reference group

## Discussion

This study validated the C-SSRS scale by examining its reliability and construct validity. The factor structure of the C-SSRS scale was evaluated by using the principal component analysis that revealed high loadings on one factor. The measure of internal consistency in this study (Cronbach’s alpha = 0.966) shows relatively adequate reliability, contrary to the Spanish version of the C-SSRS that found a two-dimensional model, including thoughts and attitudes toward suicide, with high internal consistency [76]. Three multisite, double-blind studies have evaluated the psychometric properties of the C-SSRS in adolescents and revealed high internal reliability (α = 0.73 to 0.95) and good convergent validity (r = 0.80), with well-known suicidal instruments [46]. Cultural differences between countries might have led to the nonconformity of factor loadings since the behavior and expression of emotions differ from a person to another due to the environmental influence [77].

Our study revealed a high prevalence of suicidal thoughts (28.9%) among Lebanese teenagers, higher than that found in other studies conducted in Lebanon [3, 78, 79]. A study that examined the prevalence and risk factors for suicide ideation among 5038 Lebanese adolescents showed that around 16% of participants thought of suicide [3], consistent with the rates of suicidal ideation among young people in the Eastern Mediterranean region, ranging between 13 and 17% [80]. Our results could be explained by several risk factors, including bullying, physical and sexual abuse, mental disorders and depressive symptoms [81]. Indeed, people living in societies at war and unstable environments are at high risk of developing mental health problems, such as depression, anxiety, and stress disorders, precursors of suicidal behaviors [41]. It is also important to highlight a possible underestimation of suicide in Lebanon and the Arab World since most of the religions prevailing consider it as a taboo, which might result in underreporting of suicide and the inability to collect data and investigate its correlates. Also, some tend to believe that asking about suicide will generate or stimulate suicidal thoughts or attempts in participants, but there is no evidence to support this hypothesis [39]. This study could open the door to other studies in terms of intervention in such situations, and screening of adolescents at risk.

In addition, adolescents in the Arab World aged between 15 and 24 face some similar issues and challenges confirmed the United Nations Economic and Social Commission for Western Asia and the United Nations Program on Youth, including socioeconomic status with high rate of poverty, low social inclusion, political troubles, high religion affiliation, low education level specially among females, poor access to health facilities, high rate of unemployment and lower participation in social, public and political life. These similarities between Arab countries show that risk factors for suicidal ideation in Lebanese adolescents might be applicable for other adolescents in the Arab World but further studies are needed to prove that.

In this study, factors associated with suicidal ideations in Lebanese adolescents could be identified as well: taking the suicidal ideation score as the dependent variable, showed that parents’ separation is a major factor affecting suicidal ideation (highest effect size = 0.99), followed by female gender, and younger age. As for other factors, they include decreasingly higher alcohol dependence (Beta = 0.062), psychological abuse (Beta = 0.041), child physical abuse (Beta = 0.030), social fear (Beta = 0.028), victimization/bullying score (Beta = 0.028), impulsivity (Beta = 0.028) and internet addiction (Beta = 0.010) scores.

In our study, some sociodemographic characteristics were associated with higher suicidal ideation in adolescents, such as being a female and being a child of a single parent, in agreement with other findings [82]. Female hormones may trigger mood disturbances that can create psychiatric troubles that have been shown to be linked to suicide, such as depression. Moreover, being a child of a single parent (due to divorce or death) can cause a decreased affection in adolescents, which might increase psychiatric troubles, thus leading to suicide [83]. While other studies found that younger teenagers are less exposed to suicide risk factors and less likely to commit suicide [84], our results showed that older adolescents are less prone to suicidal thoughts and suicide attempts. In fact, as adolescents mature, their ability to interpret situations and their capacity to manage problems increase, which might explain the decreased risk of suicide [85].

In our study, alcohol use disorder was also associated with higher risk of suicidal ideation. Hazardous alcohol drinking is toxic for mental health and can lead to many psychiatric disorders, including depression, and impulsive suicidal attempts [86], particularly in the younger population [28]. Lebanese adolescents might be at high risk for alcohol dependence probably because of the easy access to alcohol (in comparison with other countries), and its cheapness: in Lebanon, the law prohibiting the sale of alcohol to minors is not rigorously applied, and many bars that serve alcohol do not take into consideration the age of their customers, making it easier for the adolescents to drink alcohol [87].

The findings of our study showed that both psychological and physical child abuse are associated with higher suicidal ideation. Previous research has also linked child abuse and suicidal behaviors [88] and attempts [89] in the general population, highlighting the importance of preventing child abuse and recognizing it as a factor related to suicide [29]. While some studies showed that physical abuse is more likely to induce suicidal ideation [88], others revealed that emotional abuse is mainly implicated [90]. However, few studies could not associate emotional abuse with suicidal ideation in adolescents [91]. Additional research is needed to see whether the risk of suicidal ideation may vary with to the different forms of child abuse or not in the Lebanese community.

Our results also showed that social phobia is a risk factor for suicide, consistent with those of a previous study [92]. Social phobia can lead to suicide through several pathways, including depression [93], loneliness, and less intimacy with close friends [94]. People with social phobia tend to be more sensitive to negative evaluation and they adversely interpret equivocal social situations [95]; they also tend to avoid social interaction, have poor social skills and lesser friends [96], which may reduce the social support and encouragement they might ever receive.

Bullying and victimization were associated with higher rates of suicide, in agreement with previous studies showing that being a victim or a bully during adolescence increases the risk of suicidal ideations and attempts in adulthood [97]. Bullying can lead to suicide by affecting social connectedness [98] and decreasing self-esteem, thus leading to increased risk of suicide [99]. Research demonstrated that although bullying subtypes are related to each other, they may lead to different outcomes. Some studies showed that physical bullying was linked to higher rates of suicidal ideation than verbal bullying [100, 101], while others showed that both verbal and physical victimization increase the risk of suicidal ideation without any difference [102]. However, studies on how the severity of different types of bullying would affect suicidal ideations and attempts in Lebanon are lacking. Further studies are warranted to clarify this point.

In our study, a positive relationship was also found between impulsivity and suicidal ideation, consistent with previous findings [103]. Impulsivity is associated with other psychological processes like aggression, sabotage, unhealthy sexual behaviors, alcohol use disorder, and others [104] that can lead to suicide.

Our results demonstrated that internet addiction was associated with higher suicidal thoughts, consistent with previous findings [4]. Internet addiction has been linked to many psychiatric disorders in adolescents, decreasing their self-confidence and their ability to tolerate frustration, process information, and control their peers [105]. Also, adolescents with internet addiction reported troubles facing difficult situations [106] and had altered cognitive control that pushed them to take risky decisions [107], making them at increased risk of suicidal ideations. Moreover, internet addiction and psychiatric symptoms interact bi-directionally, thus amplifying one another, which can worsen the course of both illnesses [108].

### Clinical implications

The high prevalence of suicidal thoughts and related factors among Lebanese teenagers revealed in our study, highlights the need to raise awareness about suicide in the Lebanese population, especially among those who are at high risk of suicidal ideation and attempts. Strategies and plans for suicide prevention should be developed, in addition to enhancing the role of social organizations, educating adolescents and their parents about the importance of reducing all types of violence, and promoting early diagnosis of adolescents at high risk. Facilitating the treatment of suicidal ideation by setting affordable prices for medications and increasing financial support for these patients is also recommended.

### Limitations

This study presents some limitations. Its cross-sectional design does not allow deriving causal relationships. Since the data was collected from schools, adolescents who did not attend school were not included in the study, thus leading to a possible selection bias. The self-report questionnaire may increase the risk of non-differential information bias, thereby driving the hypotheses to null. Some of the scales used have never been validated in Lebanon; add to this that using the LSAS scale as a self-evaluation might be problematic. Finally, the relationship with drug abuse that was shown to be strongly related to suicide was not assessed. Finally, the validation process did not take into consideration the test-retest or the comparison with another scale to screen for suicidal ideation. Further research taking into account these limitations is needed. However, the relatively large sample size allows a close approximation of the findings to the general adolescent population, especially since no such studies, taking into consideration a representative sample from all regions, were previously conducted in Lebanon.

## Conclusion

This study provides insights about suicidal ideation among Lebanese adolescents and related risk factors, such as child psychological and physical abuse, alcohol use disorders, social fear, bullying and victimization, impulsivity, and internet addiction. Further studies are warranted to confirm these results and propose strategies to prevent suicidal ideation and suicide attempts in Lebanese adolescents, based on related risk factors. Authorities are also required to raise awareness in the population and encourage primary healthcare providers to screen for suicidal ideation in adolescents and identify risk factors as early as possible.

## Data Availability

All data generated or analyzed during this study are not publicly available to maintain the privacy of the individuals’ identities. The dataset supporting the conclusions is available upon request to the corresponding author.

## Abbreviations

WHO: World Health Organization
C-SSRS: Columbia-Suicide Severity Rating Scale
TASA: Treatment of Adolescent Suicide Attempters
IAT: Internet Addiction Test
ADRS: Adolescent Depression Rating Scale
AUDIT: Alcohol Use Disorders Identification Test
LSAS: Liebowitz Social Anxiety Scale
BIS-11: BARRAT Impulsiveness Scale
CASRS: Child Abuse Self-Report Scale
